# Research on Mechanical Properties and Sensitivity of a Novel Modified Double-Base Rocket Propellant Plasticized by Bu-NENA

**DOI:** 10.3390/ma15186374

**Published:** 2022-09-14

**Authors:** Shixiong Sun, Benbo Zhao, Yuan Cheng, Yunjun Luo

**Affiliations:** 1 School of Chemical Engineering and Technology, North University of China, Taiyuan 030051, China; 2 Dezhou Industrial Technology Research Institute of North University of China, Dezhou 253034, China; 3 School of Materials Science and Engineering, Beijing Institute of Technology, Beijing 100081, China

**Keywords:** Bu-NENA, extruded modified double-base propellant, high solid content, mechanical sensitivity, mechanical property

## Abstract

The research and development of rocket propellants with a high solid content and superior mechanical and security performance is urgently needed. In this paper, a novel extruded modified double-base (EMDB) rocket propellant plasticized by N-butyl-N-nitratoethyl nitramine (Bu-NENA) was prepared to overcome this challenge. The results indicated that Bu-NENA decreased the mechanical sensitivity successfully, contributing to the mechanical properties against traditional nitroglycerin (NG) based EMDB propellants, while hexogen (RDX), which is beneficial to propellant energy, was not conducive to the elongation and sensitivity of the propellants. By contrast with the blank group (NG-based EMDB propellant, R0), the elongation of the optimized propellant at −40 °C was promoted by 100% from 3.54% to 7.09%. Moreover, the β-transition temperature decreased from −33.8 °C to −38.1 °C due to superior plasticization by Bu-NENA, which represents a better toughness. The friction sensitivity dropped by 100% from 46% to 0%. Simultaneously, the height for 50% probability of explosion (*H*_50_) increased by 87.2% from 17.2 cm to 32.2 cm. The results of this research could be used to predict a potential prospect in tactical weapons.

## 1. Introduction

Propellant is the working medium of rocket motor. It can be solid or liquid [1,2]. Extruded modified double-base (EMDB) propellants are one of the most commonly used solid propellants for tactical rockets due to the advantages of low cost, high production efficiency, mature process, good repeatability between batches and so on [3,4]. Nitrocellulose (NC) plasticized by nitroglycerin (NG) is the most commonly used binder of this kind of propellant. Aluminum (Al) powder and nitroamine explosives are also applied, which is attributed to their high energy density. The theoretical specific impulse (*I*_sp_) of propellants made up of NC/NG/Al/hexogen (RDX) or octogen (HMX), which is the typical formula, can reach up to 260 s. Meanwhile, the density can be as high as 1.70 g/cm^3^. The good comprehensive properties promoting this kind of propellant mean that they are widely used in rocket motors [5]. However, NG is extremely sensitive to mechanical stimuli which results in high mechanical sensitivity for NG-based EMDB propellants [6,7,8]. Especially when the solid content is up to 53% (calculated by the total amount of RDX and Al), the friction sensitivity of propellants can reach more than 50%, and the *H*_50_ can be lower than 15 cm, which affects their application in weapons [9]. Explosion accidents caused by sensitive properties happen from time-to-time, which can cause serious consequences for human lives and weapon platforms. Therefore, the development of modern weapons has an urgent need for low-sensitivity EMDB propellants. Additionally, the tensile performance of NG-based EMDB propellants with a high solid content is very poor. Elongation at −40 °C can be lower than 3%, which can readily cause structural damage and further accidents [10]. Hence, using insensitive plasticizers with high energy instead of NG has become an important subject in the field of solid rocket propellant research to reduce the mechanical sensitivity and improve the mechanical properties of propellants [11,12].

N-butyl-N-nitratoethyl nitramine (Bu-NENA) is a nitrate ester energetic plasticizer which was first synthesized by Blomquist and Fiedorik at 1949 [13]. The n-butyl group makes Bu-NENA have a better flexibility than NG. Meanwhile, the nitramine and nitrate ester groups can give it an appropriate energy level [14]. Reasonably distributed energetic groups make Bu-NENA less sensitive. Its friction and impact sensitivity are 0 and 110 cm (*H*_50_), respectively. In addition, it has good thermochemical stability. When used in gun propellants, which have some similarity with rocket propellants in their composition, while their configuration and performance are quite different, it can reduce the sensitivity of the products significantly, and improve the process properties and low-temperature mechanical properties [15]. In short, the comprehensive properties of many energetic composite materials could be improved with the use of Bu-NENA [16,17]. Therefore, it is of great significance to study the application of Bu-NENA in EMDB propellants with a high solid content.

Inspired by the beneficial effects of Bu-NENA on gun propellants [16], we previously introduced it into a double-base rocket propellant [18], which was the original type of solid propellant and had no solid fillers. It showed great potential for application in rocket propellants. In view of the urgent demand of modern weapon platforms for low sensitivity propellants, a novel series of EMDB rocket propellants with a high solid content were prepared by using Bu-NENA with good flexibility and low sensitivity as a plasticizer. The influence of the Bu-NENA and RDX contents on the sensitivity and mechanical properties of EMDB propellants was studied. The results showed that the comprehensive performance of EMDB propellant could be improved due to the good physical and chemical properties of Bu-NENA.

## 2. Materials and Methods

### 2.1. Materials

NC (12.0% N) and nitroglycerin (NG) were obtained from Shanxi Northern Xing’an Chemical Industry Co., Ltd., Taiyuan, China. Bu-NENA was obtained from Liming Research Institute of Chemical Industry, Luoyang, China. Hexogen (RDX) of 72 μm (from supplier) was obtained from Gansu Silver Light Chemical Industry Group Co., Ltd., Baiyin, China. Spherical Al of 3 μm was obtained from Changyuan Mingyu Aluminium Industry Co., Ltd., Xinxiang, China.

### 2.2. Propellant Preparation

The novel EMDB propellants plasticized by Bu-NENA with thickness ca. 2 mm were prepared through the traditional solvent-free method. The preparation process was similar to that described in the literature [19]. EMDB propellant plasticized by NG was also prepared as a control. The detailed chemical ingredients of the prepared propellants are shown in Table 1. The additives were organic lead-salt, organic copper-salt and carbon black, vaseline and N, N’-dimethyl carbanilide. It should be noted that the NC/Bu-NENA adhesive decreases with the increase in RDX content for R series EMDB propellants. The mass ratio of NC/Bu-NENA remained 43/57 due to the results of previous exploration experiments in double-base propellant in which there were no solid fillers [18].

### 2.3. Characterization and Analysis

#### 2.3.1. Mechanical Properties Test

The propellant sheet was cut into dumbbell-shaped test specimens. The propellant mechanical properties were conducted on the AGS-J Electronic Universal Testing Machine (Shimadzu Corporation, Kyoto, Japan) with the China Military Standard GJB 770B-2005 413.1. The conditions were: temperature −40 °C, 20 °C and 50 °C; and tensile rate 10 mm/min. The testing apparatus was equipped with a high–low temperature test box which could provide the required ambient temperature. The propellant specimens were placed in the test box for 40 min before being tested.

#### 2.3.2. Dynamic Mechanical Properties Test

Dynamic mechanical property analysis was conducted using a dynamic thermomechanical analyzer (DMA, METTLER TOLEDO, DMA/SDTA 861e, Zurich, Switzerland) in shear mode. The conditions were: temperature −120~120 °C; heating rate 3 K/min; dynamic force 5 N; frequency 1 Hz; amplitude 5 μm; and sample size 5 mm × 5 mm× 2 mm.

#### 2.3.3. Sensitivity Measurement

The friction sensitivity was determined according to the China Military Standard GJB 770B-2005 601.2 using a pendulum friction apparatus (Beijing nachen Technology Co., Ltd., Beijing, China). The conditions were: pendulum weight 1.5 kg; swaying angle 66 deg; pressure 2.45 MPa; and sample mass 20 ± 1 mg. The initiation probability P was obtained from 50 trials. The impact sensitivity was determined according to the China Military Standard GJB 770B-2005 602.1 on a drop-hammer apparatus (Beijing nachen Technology Co., Ltd., Beijing, China) using an up-and-down method. The conditions were: sample mass 30 mg; and hammer weight 2 kg. Based on 25 go/no-go trials, the height for 50% probability of explosion (*H*_50_) could be calculated.

## 3. Results and Discussion

### 3.1. Mechanical Properties of EMDB Propellants

**Figure 1:**
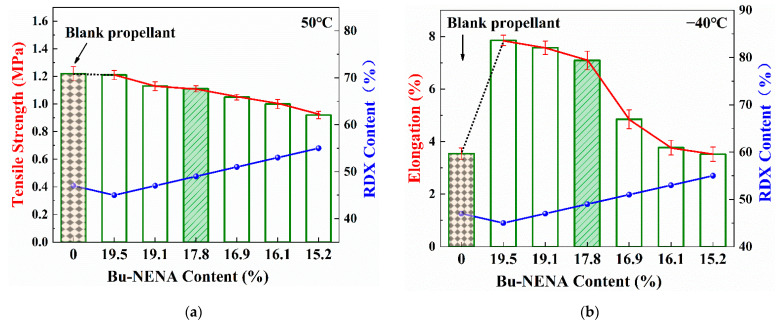
Mechanical properties of EMDB propellant: (**a**) Tensile strength of EMDB propellant at 50 °C; (**b**) Elongation of EMDB propellant at −40 °C.

Table S1 in the supporting information shows the propellants mechanical properties. In order to obtain the influence law of the Bu-NENA on the EMDB propellants mechanical properties more intuitively, the curves of the tensile strength at 50 °C and elongation at −40 °C with Bu-NENA and RDX content are shown in Figure 1.

It could be found from Table S1 and Figure 1 that the tensile strength of the prepared propellants reduced slightly compared with the referenced propellant. This was mainly caused by two reasons. On the one hand, it has been proved that Bu-NENA can plasticize NC more readily, which can decrease the intermolecular force [20], enhancing the molecular mobility of NC. This is a negative factor for the tensile strength of propellants. On the other hand, Bu-NENA increased the fluidity of propellant adhesion [21]. The defects could be decreased, and the molding quality could be promoted, which is a positive factor for its tensile strength. The first factor is more influential between the two reasons. Therefore, the tensile strength of the prepared propellants was slightly lower than that of the control, while the elongation of the prepared propellants could be significantly improved over that of the blank group. This is because both of the two kinds of effects mentioned above could have contributed to the elongation of the prepared propellants.

Moreover, both the tensile strength and the elongation of the prepared propellants reduced with the increase in the RDX content. This was mainly caused by the relatively poor interface character between the RDX and double-base adhesive [22]. The insufficient wettability lowered the adhesion between them, which may have caused the formation of microscopic defects in the EMDB propellant with a high solid content. Even if there are no defects in the propellant, this could readily occur when subjected to an external force due to the low adhesion. These defects make propellants break earlier. Consequently, both the tensile strength and the elongation of the prepared propellants reduced along with the increase in RDX content. Additionally, the NC/Bu-NENA adhesive decreased with the increase in RDX content. The percentage reduction reduced the deformation capacity of the propellant, which could also be responsible for the decline in elongation. It is worth noting that the mass ratio of NC/Bu-NENA remained 43/57 in the prepared propellants. Therefore, the reduction in Bu-NENA content was not related to the propellant’s mechanical properties directly.

As mentioned above, it could be concluded that a lower content of RDX may endow better mechanical properties to propellants. However, it has to be noted that a high RDX content is beneficial for improving the *I*_sp_ of propellants, as shown in Table 2 (calculated due to the NASA 273 software based on the minimum free energy method). As a result, R3 could be considered the best among all of the prepared propellants, as it exhibited both improved mechanical properties and a satisfactory theoretical specific impulse. Quantitatively, the elongation of the R3 propellant at −40 °C increased by 100% from 3.54% to 7.09% compared with the control, and the tensile strength at 50 °C of the R3 propellant was 1.11 MPa, which is the same level as the control.

### 3.2. Dynamic Mechanical Properties of EMDB Propellants

**Figure 2:**
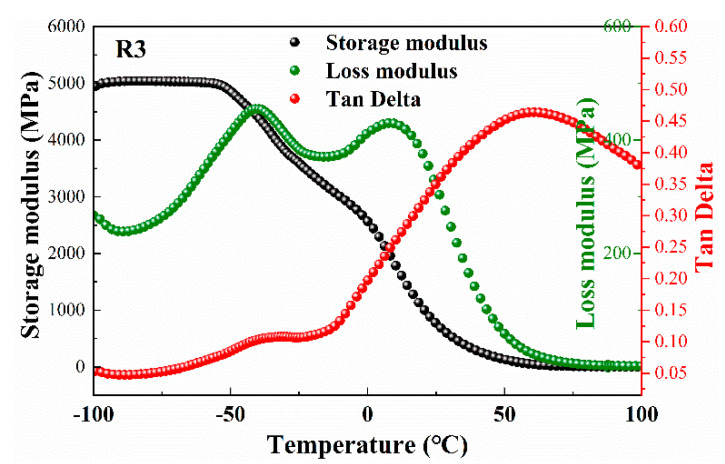
The *E*′, *E*″ and Tan*δ* curves of R3 propellant.

Dynamic thermomechanical analysis was conducted for further understanding of the influence of the Bu-NENA and RDX on the properties of the propellants. The DMA curves of the R3 propellant are shown in Figure 2, and the DMA curves of the other prepared propellants, which are similar to that of R3, are given in Figure S1 in the supplementary files. The transformation of the dynamic storage modulus (*E*′), dynamic loss modulus (*E*′) and Tan*δ* of the R3 propellant against the temperature are shown in Figure 2. *E*′ represents the elastic properties of the propellant, *E*” represents viscous properties, and Tan*δ* represents damping of the propellant (*E*″/*E*′) [23]. As shown in Figure 2, the storage modulus *E*′ exhibited two obvious reductions at temperatures ca. −50.0 °C and 0 °C (onset temperature), which were considered to be related to the softening of the propellant. Furthermore, there are two peaks in the *E*” curve at the same region. This is due to the presence of the two thermal transitions for the NC/Bu-NENA adhesion. The β-transition was in the range of −50~0 °C, while the α-transition appeared in the range of 0~50 °C. The β-transition is attributed to the single-bond rotation, stretching and bending of side groups, cooperative motion between the side groups of NC and Bu-NENA, and so on. The α-transition was considered to be associated with some slightly larger motion unit-like segment in the NC molecular backbone. The peak temperature of the two transitions (*T*_α_ and *T*_β_) are important for the mechanical performance of propellants, which is focused on by many researchers. According to the literature [9], there are at least a few methods of glass-transition determination from the results obtained by dynamic mechanical analysis, among which the peak temperature of Tan*δ* is preferred due to its relatively stable value (Tan*δ* cannot be disturbed by test conditions readily) [24]. Accordingly, the temperature of the β-transition and α-transition were −38.1 °C and 58.2 °C, respectively.

**Figure 3:**
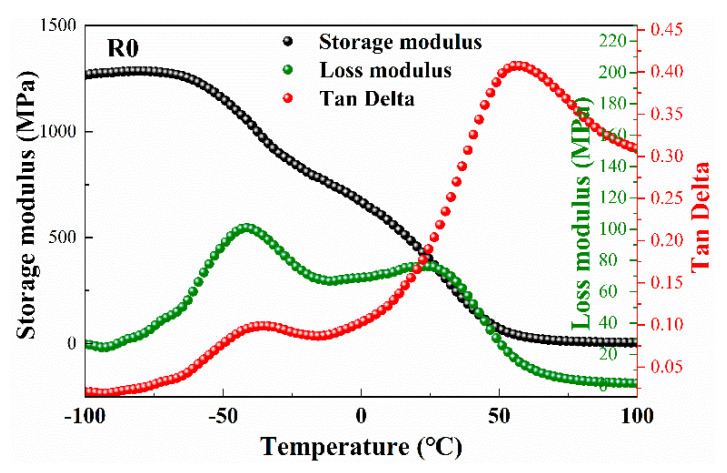
The *E*′, *E*″ and Tan*δ* curves of R0 propellant.

The *E*′, *E*″ and Tan*δ* plots of the blank EMDB propellant (R0) are shown in Figure 3. It is obvious that the trends of change for the three curves were similar with that of the R3 propellant plasticized by Bu-NENA, while the transition temperatures differed. The *T*_β_ of the R3 was −38.1 °C, which is lower than that of R0 (−33.8 °C). The difference mainly arose from the more obvious reduction in Bu-NENA on the intermolecular forces between the adhesive molecules, which endowed Bu-NENA with a better plasticization effort. The motion of side groups in the NC molecule of R3 propellant could occur more readily than in R0 propellant, which is to say the activation energy of molecular motion of R0 is lower. Furthermore, the elastic deformation energy of R0 is more easily dissipated and converted into heat [25]. Therefore, the β-transition temperature of R3 decreased by a couple of degrees. As for the α-transition, the *T*_α_ of the R3 propellant was similar to that of the R0 propellant. This might have been caused by the influence of RDX fillers on the double-base adhesive. There are inducing forces between the nitramine groups in RDX and the nitrate ester group in the adhesive. This kind of intermolecular force is less affected by temperature compared with the interaction force between NC molecules such as hydrogen bonds. The difference in the mentioned interaction force between the R3 and R0 propellants was weakened due to the temperature rising. However, the RDX content in R3 was slightly higher than that in R0, which was not conducive to the motion of adhesive molecules in the R3 propellant. Accordingly, the *T*_α_ of the R3 propellant was similar with that of the R0 propellant.

**Figure 4:**
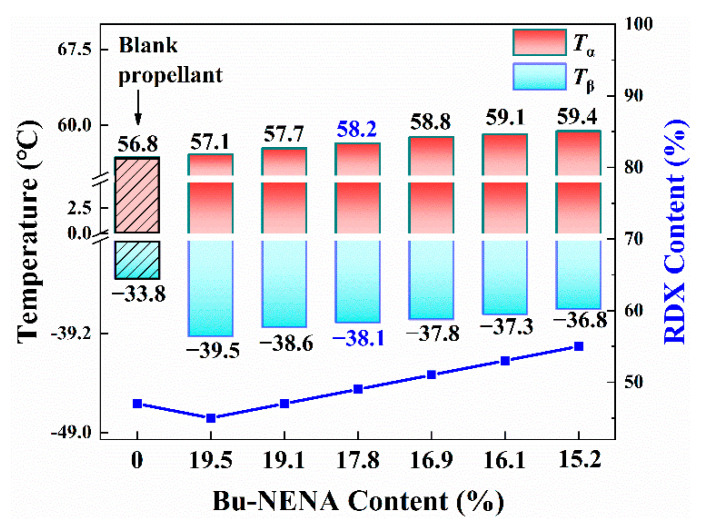
The values of *T*_α_ and *T*_β_ of prepared propellants.

In order to obtain the effect of RDX on the glass transition temperature of propellants more intuitively, the values of *T*_α_ and *T*_β_ are given in Figure 4 and Table S2 in the supporting information. *T*_β_ is a parameter of the adhesive system of propellants, which is related to the molecular motion of the side group in NC. A lower *T*_β_ means better molecular mobility of the NC/Bu-NENA system. Additionally, deformation could occur more readily for adhesive systems at low temperature. It is obvious that both *T*_α_ and *T*_β_ increased along with the enrichment of the RDX concentration. It may have been mainly caused by the hindrance to molecular motion of the adhesive from RDX as mentioned above.

**Figure 5:**
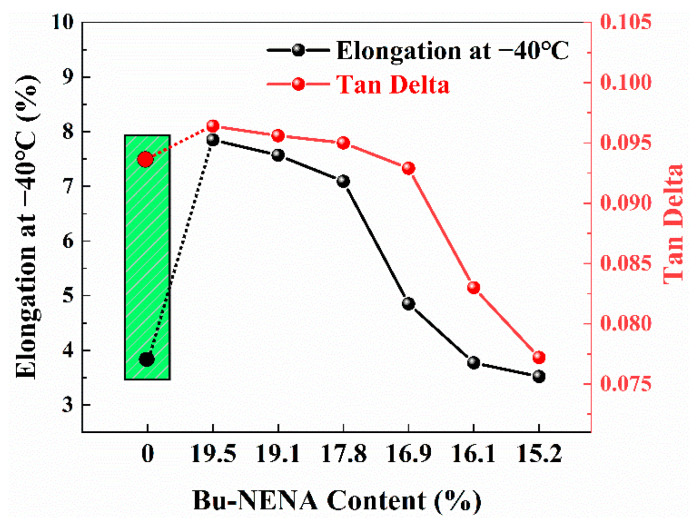
The elongation and Tan*δ* of R series of propellants.

What is interesting is that we found that there was some relationship between elongation at −40 °C and Tan*δ* as shown in Figure 5. This might be associated with the origin of Tan*δ*. Tan*δ* is calculated from *E*″/*E*′ which represents the ratio of energy that can be dissipated into heat from stored energy [26], that is, the so-called damping or toughness. Both the damping and elongation depend on the intermolecular interaction characteristics of propellants during deformation. Thereby, the change law of Tan*δ* was consistent with the elongation of the prepared propellants. However, there was no such relationship between the prepared propellants and the blank sample as shown in Figure 5. It may have been caused by the difference between the adhesive of the two kinds of EMDB propellant since the plasticization effort of Bu-NENA was significantly better than that of NG.

### 3.3. Mechanical Sensitivity

**Figure 6:**
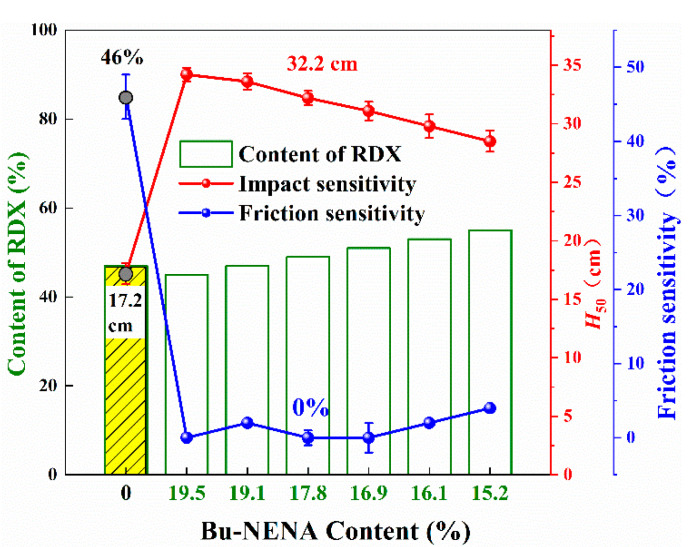
The friction and impact sensitivity of R series of propellants.

The friction and impact sensitivity of the prepared propellants are shown in Figure 6. It can be seen from Figure 6 that the mechanical sensitivity of the prepared propellants was significantly lower than that of the R0 propellant. Many reasons may contribute to the reduction in sensitivity. First of all, Bu-NENA is insensitive to mechanical stimuli [12], as it can absorb the energy transmitted by external stimulation effectively [27,28]. Secondly, Bu-NENA can plasticize NC and enhance the molecular motion ability of the adhesive system. Under mechanical stimulation, the adhesive can produce a continuous plastic flow forming a buffer protection for the solid fillers and reduce the probability of hot spot formation resulting from friction and the extrusion of fillers [29,30]. At the same time, the probability of adiabatic compression hot spot formation could be reduced due to the enhancement of adhesive movement ability [31]. Thirdly, the oxygen balance of the prepared propellants was slightly lower than that of the control, which could also reduce the sensitivity of propellants [32].

It is worth noting that the mechanical sensitivity increased slightly with the RDX content increasing, which may have been caused by the physical and chemical properties of RDX. The high mechanical sensitivity of RDX is determined by its own characteristics and it could be exacerbated due to the irregular shape and crystal defects in industrial RDX products [8]. The probability of compression and shear hot spot formation increased along with RDX content increasing in the prepared propellants. Therefore, the corresponding friction and impact sensitivity increased slightly as shown in Figure 6. Similarly to Section 3.1, it was expected to find a balance between excellent mechanical sensitivity and a satisfactory theoretical specific impulse. As shown, the friction sensitivity of the R3 propellant reduced 100% from 46% to 0% and the *H*_50_ increased by 87.2% from 17.2 cm to 32.2 cm. At the same time, the increase in RDX content was beneficial to the *I*_sp_ due to its high energy density nature. Thereby, the R3 was considered to be the optimal formula, which has potential applications.

## 4. Conclusions

A novel EMDB propellant plasticized by Bu-NENA with a high solid content was prepared, and the mechanical properties and mechanical sensitivity were evaluated. The main conclusions of this work are as follows:

(1) NC was well plasticized by Bu-NENA. The intermolecular forces were decreased, and the molecular mobility of NC was enhanced. Therefore, the β-transition temperature decreased and elongation at −40 °C was promoted. By contrast with the blank group, the *T*_β_ for the R3 propellant, which is considered to be a better formula, decreased from −33.8 °C to −38.1 °C, and the elongation at −40 °C increased by 100% from 3.54% to 7.09%. The tensile strength of the R3 propellant at 50 °C remained at the same level as that of the control due to the contribution from RDX.

(2) Bu-NENA was insensitive to mechanical stimuli. It could absorb the energy transmitted by external stimulation effectively and reduce internal defects, resulting in the mechanical sensitivity of the prepared propellants being lower than that of the control, while the RDX, which is beneficial to propellant energy, was not conducive to the mechanical sensitivity of propellant. The friction sensitivity of R3 dropped by 100% from 46% to 0%, whilst simultaneously its *H*_50_ increased by 87.2% from 17.2 cm to 32.2 cm.

In summary, the low-temperature mechanical properties and sensitivities were improved significantly for the novel EMDB propellant, and it could be predicted to be a potential prospect in tactical weapons. Since the two drawbacks, which are of concern to many researchers, were largely improved upon, we will be devoted to the study of the combustion and experimental specific impulse performance of EMDB propellants in future work.

## Figures and Tables

**Table 1:** The composition of EMDB propellant plasticized by Bu-NENA (wt. %).

Sample	NC/%	NG/%	Bu-NENA/%	RDX/%	Al/%	Additives/%
R0	21.4	22		47	6	3.6
R1	25.9		19.5	45	6	3.6
R2	24.3		19.1	47	6	3.6
R3	23.6		17.8	49	6	3.6
R4	22.5		16.9	51	6	3.6
R5	21.3		16.1	53	6	3.6
R6	20.2		15.2	55	6	3.6

**Table 2:** The theoretical specific impulse of prepared propellants.

Sample	OB ^1^	ρ/cm^−3^	*I*_sp_/s
R1	0.4467	1.634	257.4
R2	0.4486	1.640	258.2
R3	0.4538	1.651	258.9
R4	0.4577	1.659	259.6
R5	0.4613	1.667	260.4
R6	0.4653	1.676	261.1

^1^ OB is the oxygen balance.

## Data Availability

Not applicable.

## Supplementary Materials

The following supporting information can be downloaded at: https://www.mdpi.com/article/10.3390/ma15186374/s1, Figure S1: The *E*′, *E*″ and Tan*δ* plots of the prepared propellants. Table S1: The mechanical properties of the EMDB propellant plasticized by Bu-NENA. Table S2: The transition temperature of the EMDB propellant.
