# Supplementary material for: Research on Mechanical Properties and Sensitivity of a Novel Modified Double-Base Rocket Propellant Plasticized by Bu-NENA
Source: Materials (Basel). 2022 Sep 14;15(18):6374. doi: 10.3390/ma15186374 (PMC9504536; doi:10.3390/ma15186374)
Supplement: Supplementary file 1 [file materials-15-06374-s001.zip › materials-1872118-supplementary.pdf]

Supporting Information

Research on Mechanical Properties and Sensitivity of a Novel Modified Double-Base Rocket Propellant Plasticized by Bu-NENA

The Authors:

Shixiong Sun [1,2], Benbo Zhao [1,2], Yuan Cheng [1,*] and Yujun Luo [3,*]

Corresponding Author: Prof. Benbo Zhao; Prof. Yunjun Luo

Address:

1 School of Chemical Engineering and Technology, North University of China, Taiyuan 030051, China

2 Dezhou Industrial Technology Research Institute of North University of China, Dezhou 253034, China

3 School of Materials Science and Engineering, Beijing Institute of Technology, 5 South Zhongguancun Street, Beijing 100081, China.

Tel.: +86-10-3922315; +86-10-68913698

E-mail: zhaobenbo@163.com (B. Zhao); yjluo@bit.edu.cn (Y. Luo).

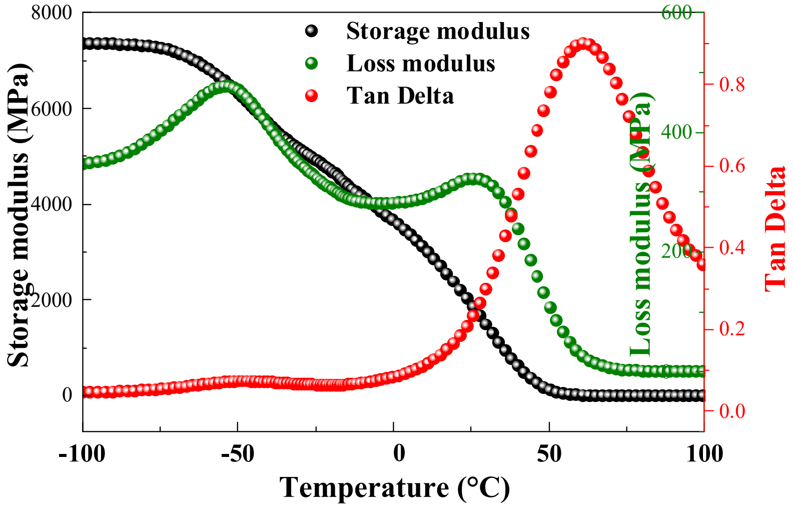
Storage modulus
Loss modulus
Tan Delta
Storage modulus (MPa)
Loss modulus (MPa)
Tan Delta
Temperature (°C)

(a)

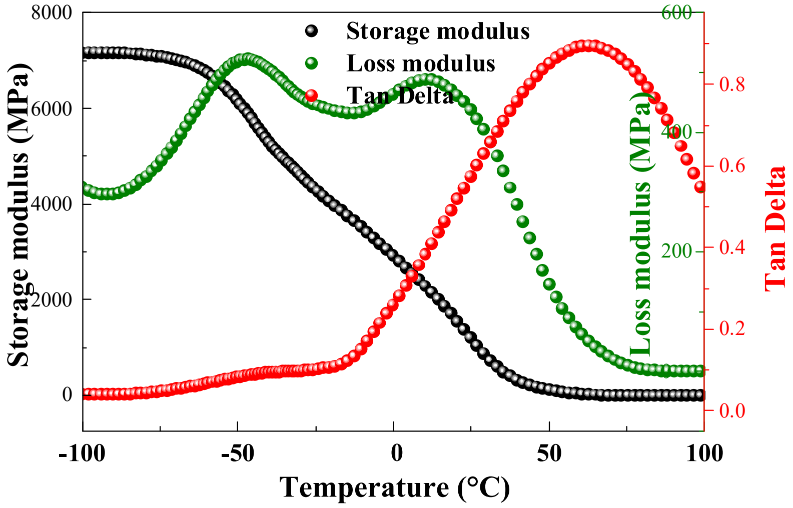
Storage modulus
Loss modulus
Tan Delta
Storage modulus (MPa)
Loss modulus (MPa)
Tan Delta
Temperature (°C)

(b)

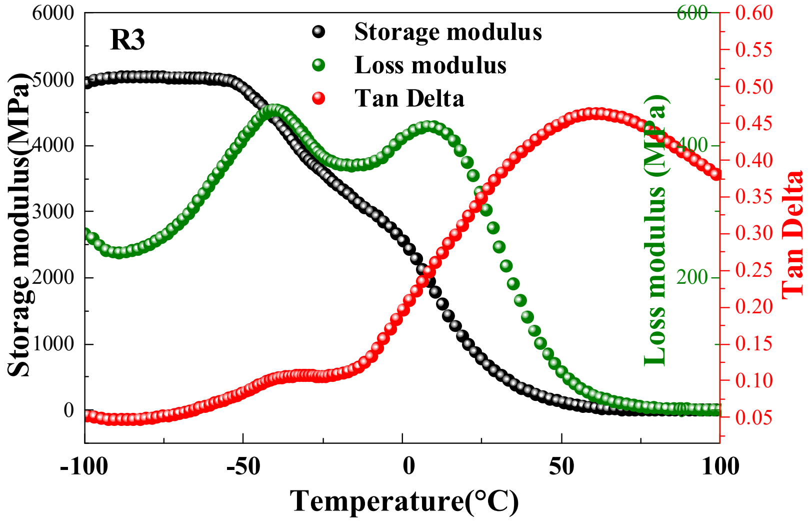
R3
Storage modulus
Loss modulus
Tan Delta
Storage modulus(MPa)
Loss modulus (MPa)
Tan Delta
Temperature(°C)

(c)

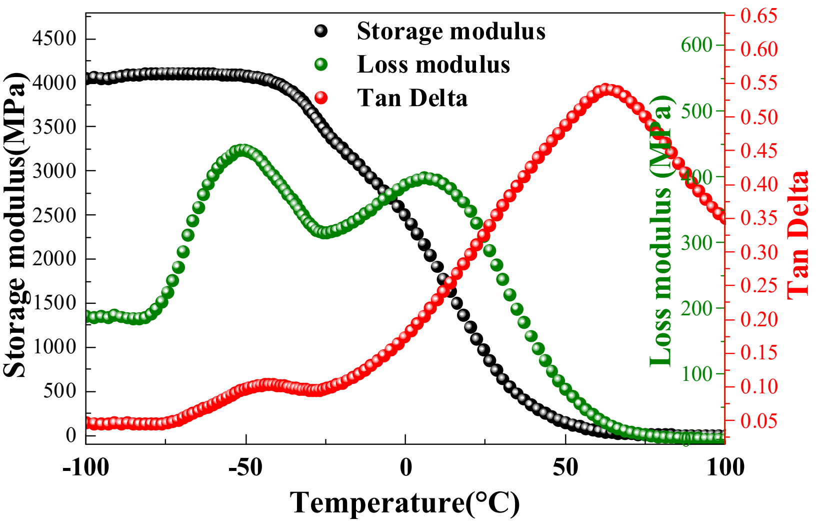
Storage modulus
Loss modulus
Tan Delta
Storage modulus(MPa)
Loss modulus (MPa)
Tan Delta
Temperature(°C)
-100
-50
0
50
100

(d)

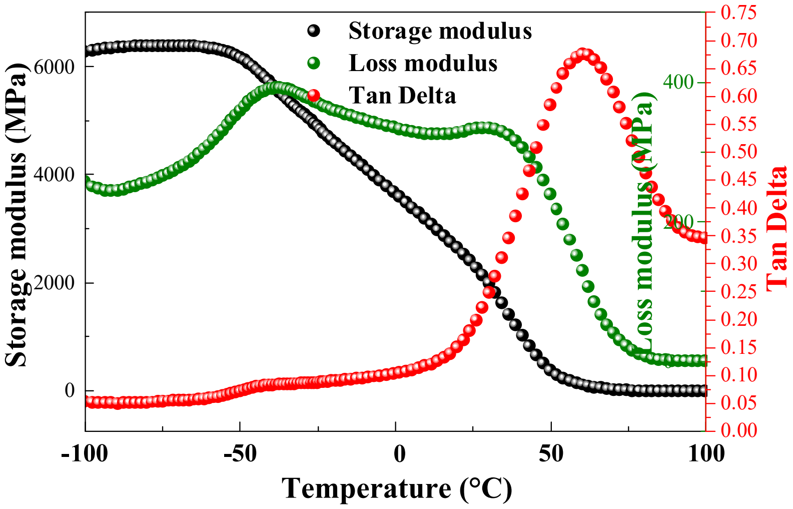
Storage modulus
Loss modulus
Tan Delta
Storage modulus (MPa)
Loss modulus (MPa)
Tan Delta
Temperature (°C)
-100
-50
0
50
100

(e)

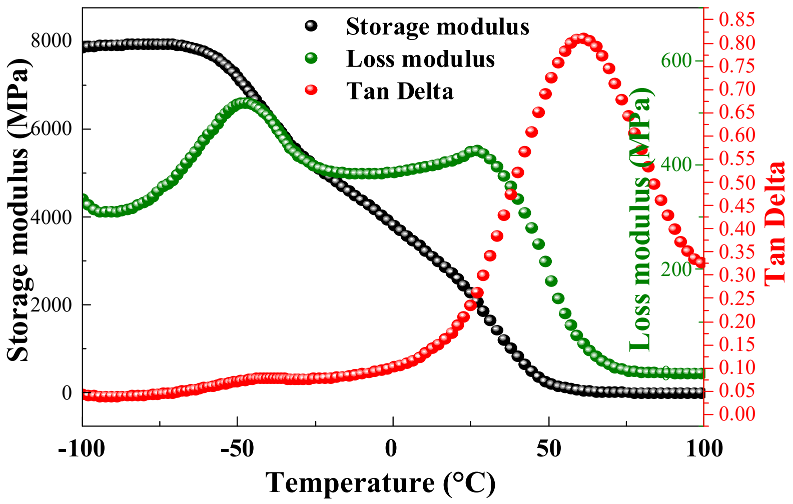
Storage modulus
Loss modulus
Tan Delta
Storage modulus (MPa)
Loss modulus (MPa)
Tan Delta
Temperature (°C)
-100
-50
0
50
100

(f)

Figure S1. The *E*', *E*'' and Tan*δ* curves of R series propellants. (**a**) R1; (**b**) R2; (**c**)R3; (**d**) R4 (**e**) R5; (**f**)R6

Table S1. The mechanical properties of EMDB propellant plasticized by Bu-NENA.

**Sample**	**−40°C**		**+20°C**		**+50°C**	
	σ_m	ε_b	σ_m	ε_b	σ_m	ε_b
R0	19.6	**3.54**	7.23	5.21	**1.22**	10.9
R1	21.0	7.85	5.83	11. 67	1.21	25.97
R2	20.6	7.57	5.28	10.87	1.13	23.03
R3	20.0	7.09	5.20	9.96	1.11	22.74
R4	19.6	4.85	4.86	8.69	1.05	18.52
R5	19.0	3.77	4.41	7.33	1.00	16.85
R6	18.1	3.52	4.22	5.99	0.92	15.82

Table S2. The transition temperature of EMDB propellant.

**Sample/°C**	T_α/°C	T_β/°C
R0	56.8	−33.8
R1	57.1	−39.5
R2	57.7	−38.6
R3	58.2	−38.1
R4	58.8	−37.8
R5	59.1	−37.3
R6	59.4	−36.8
